# Genetic dissociation of three antigenic genes in *Plasmodium ovale curtisi* and *Plasmodium ovale wallikeri*

**DOI:** 10.1371/journal.pone.0217795

**Published:** 2019-06-06

**Authors:** Naowarat Saralamba, Francois Nosten, Colin J. Sutherland, Ana Paula Arez, Georges Snounou, Nicholas J. White, Nicholas P. J. Day, Arjen M. Dondorp, Mallika Imwong

**Affiliations:** 1 Department of Molecular Tropical Medicine and Genetics, Faculty of Tropical Medicine, Mahidol University, Bangkok, Thailand; 2 Mahidol-Oxford Tropical Medicine Research Unit, Faculty of Tropical Medicine, Mahidol University, Bangkok, Thailand; 3 Shoklo Malaria Research Unit, Mahidol-Oxford Tropical Medicine Research Unit, Bangkok, Thailand; 4 Immunology Unit, Department of Infectious and Tropical Diseases, London School of Hygiene and Tropical Medicine, London, United Kingdom; 5 Global Health and Tropical Medicine, GHTM, Instituto de Higiene e Medicina Tropical, IHMT, Universidade Nova de Lisboa, UNL, Lisbon, Portugal; 6 CEA-Université Paris Sud 11-INSERM U1184, Immunology of Viral Infections and Autoimmune Diseases (IMVA), IDMIT Department, IBFJ, DRF, Fontenay-aux-Roses, Paris, France; 7 Centre for Tropical Medicine and Global Health, Nuffield Department of Medicine, University of Oxford, Oxford, United Kingdom; Universidade Federal de Minas Gerais, BRAZIL

## Abstract

*Plasmodium ovale curtisi* and *Plasmodium ovale wallikeri* are two sympatric human malaria species prevalent in Africa, Asia and Oceania. The reported prevalence of both *P*. *ovale* spp. was relatively low compared to other malaria species, but more sensitive molecular detection techniques have shown that asymptomatic low-density infections are more common than previously thought. Whole genome sequencing of both *P*. *ovale* spp. revealed genetic dissociation between *P*. *ovale curtisi* and *P*. *ovale wallikeri* suggesting a species barrier. In this study we further evaluate such a barrier by assessing polymorphisms in the genes of three vaccine candidate surface protein: *circumsporozoite protein/ thrombospondin-related anonymous-related protein* (*ctrp*), *circumsporozoite surface protein* (*csp*) and *merozoite surface protein 1* (*msp1*). The complete coding sequence of *ctrp* and *csp*, and a partial fragment of *msp1* were isolated from 25 *P*. *ovale* isolates and compared to previously reported reference sequences. A low level of nucleotide diversity (Pi = 0.02–0.10) was observed in all three genes. Various sizes of tandem repeats were observed in all *ctrp*, *csp* and *msp1* genes. Both tandem repeat unit and nucleotide polymorphism in all three genes exhibited clear dimorphism between *P*. *ovale curtisi* and *P*. *ovale wallikeri*, supporting evidence of non-recombination between these two species.

## Introduction

*Plasmodium ovale curtisi* and *Plasmodium ovale walllikeri* are two sympatric species of malaria parasites found across many malaria endemic countries in Africa, Asia and Oceania [1–3]. Although morphological features of *P*. *ovale curtisi* and *P*. *ovale wallkeri* are indistinguishable, these two *P*. *ovale* species are genetically distinct, and there is evidence of differences in latency and clinical presentation [4–6]. Nuclear genome sequences of *P*. *ovale curtisi* and *P*. *ovale wallikeri* were recently reported and revealed different expansion in some gene families [7]. Currently the target genes used for discriminating between *P*. *ovale curtisi* and *P*. *ovale wallikeri* are the *SSU rRNA* gene [8], *tryptophan rich antigen* (*potra*) [9], *reticulocyte-binding protein 2* (*porbp2*) [9], and some sexual stage proteins [9]. Sequence polymorphisms in the cell-surface associated proteins that are candidate targets for vaccine development have only been studied rarely. The current study assessed genetic diversity in a highly polymorphic region of the blood stage merozoite surface protein gene *msp1*, and in two genes encoding sexual stage and sporozoite proteins, *ctrp* and *csp* respectively, in *P*. *ovale curtisi* and *P*. *ovale wallikeri*.

CTRP is a member of the micronemal and cell-surface associated proteins. In *P*. *falciparum* disruption of the *ctrp* gene prevents oocyst development in the anopheline mosquito [10], indicating that CTRP is important for mosquito midgut development. For this reason CTRP has been proposed as a transmissions-blocking vaccine candidate. CSP is the major surface protein on the *Plasmodium* sporozoite. It is a candidate target for pre-erythrocytic stage vaccine development. Genetic polymorphism within the *csp* gene have been investigated in most human malaria species including *P*. *falciparum* [11, 12], *P*. *vivax* [13, 14], *P*. *malariae* [15, 16], and *P*. *knowlesi* [17], but not in *P*. *ovale*. MSP1 is one of the predominant antigen expressed in the erythrocytic stage of *Plasmodium* spp. The *msp1* gene is highly polymorphic and has been well characterized in *P*. *falciparum* [18, 19] and *P*. *vivax* [13, 14]. A study of *P*. *ovale* isolates from Thailand revealed low diversity in the *msp1* gene [20].

The current study evaluates sequence diversity of *ctrp*, *csp* and *msp1*, in a wider collection of *P*. *ovale* isolates collected from Thailand and African countries. Assessing diversity in these surface proteins is important for defining vaccine candidates, and to further assess the species barrier between *P*. *ovale curtisi* and *P*. *ovale wallikeri*. In the current era of malaria elimination, the better understanding of *P*. *ovale curtisi* and *P*. *ovale wallikeri* is essential to ensure success against all human malaria species.

## Materials and methods

### Samples

Twenty-five samples of *P*. *ovale* (14 *P*. *ovale wallikeri* and 11 *P*. *ovale curtisi*) were collected from Thailand and African countries during 1995–2010 (S1 Table). All samples were obtained from patients enrolled in previous studies who gave written informed consent to blood sampling. Parasitaemia of these samples varied from 1 per 500 WBC to 198 per 500 WBC. The protocol for this study was reviewed and approved (reference number MUTM2001-049-04) by the ethics committee of the Faculty of Tropical Medicine, Mahidol University, Thailand. Genomic DNA of all samples was confirmed for the present of *P*. *ovale*. Nested PCR of the *SSU rRNA* gene was performed with primer rPLU1/rPLU5 in the primary reaction and with primer rOVA1/rPLU2 in the secondary reaction [21]. A nested PCR protocol based on the linker region of *dhfr-ts* gene was applied with primer Pla-DHFR-F/Pla-TS-R in the primary reaction and with primer PO-Lin-F/PO-Lin-R in the secondary reaction [22]. In addition, a semi-nested PCR of *potra* gene was performed with a primer specific to both *P*. *ovale* spp. (PoTRA-F/PoTRA rev3) in the primary reaction and with specific *P*. *ovale curtisi* (PoTRA-F/PocTRA-R) and *P*. *ovale wallikeri* (PoTRA-F/PowTRA-R) primers in the secondary reaction [23].

### Isolation of *poctrp* and *pocsp* gene

Specific primers targeting *poctrp* and *pocsp* genes were designed to obtain the full length of those two gene sequences (Table 1). A semi-nested PCR approach was used for amplification of each fragment with PCR conditions as presented in Table 1. All PCR reactions were performed with 10 mM Tris-HCl, pH 8.3, 50 mM KCl, 2 mM MgCl_2_, 125 μM dNTPs, 250 nM of each primer and 4 unit of Taq Polymerase (Kapa biosystems, USA). PCR products were then purified by Gel/PCR purification kit (Favogen, Taiwan), before being submitted for DNA sequencing.

**Table 1:** Primer sequences and PCR conditions for isolation of *poctrp*, *pocsp* and *pomsp1* genes.

				No. of PCR cycle		
Target gene	Primer name	Sequences (5' to 3')	Annealing temperature (oC)	Nest 1	Nest 2	Product size (bp)
*Poctrp*	OCSP_F120	CGTAGGAGCTGGGAATCAAG	56	30		1,500
	OCSP_R15k	TTTCCCCCGATTCAATATCA				
	OCSP_F120	CGTAGGAGCTGGGAATCAAG	58		35	980
	OCSP_R10k	ACTGCATGAGTTGCAAAACG				
	OCSP_F120	CGTAGGAGCTGGGAATCAAG	56	30		1,500
	OCSP_R15k	TTTCCCCCGATTCAATATCA				
	OCSP_F700	AGCTCCATGAAATGGGTTTG	58		35	800
	OCSP_R15k	TTTCCCCCGATTCAATATCA				
	OCSP_F700	AGCTCCATGAAATGGGTTTG	56	30		1,300
	OCSP_R20k	CCCACATGCACTGAATTACG				
	OCSP_F12k	ACCGGGAACGCATATTGTAG	58		35	750
	OCSP_R20k	CCCACATGCACTGAATTACG				
	OCSP_F16k	GGGAAAATCCAGATTCGTCT	56	30		1,400
	OCSP_R30k	ATAACCGAAGCACCAACACC				
	OCSP_F18k	TTTTACACCGTGCACAAACG	58		35	1,160
	OCSP_R30k	ATAACCGAAGCACCAACACC				
	OCSP_F5start	AAATGCGAGGCAAAAGACAAA	57	30		1,500
	OCSP_R15k	TTTCCCCCGATTCAATATCA				
	OCSP_F5start	AAATGCGAGGCAAAAGACAAA	59		35	1,000
	OCSP_R10k	ACTGCATGAGTTGCAAAACG				
	OCSP_F2700	TTTCTGATAAGGCAAGTTACGAGA	57	30		1,600
	OCSP_R4300	CAAATCTGTATTTGATTTTCCTTCAA				
	OCSP_F2700	TTTCTGATAAGGCAAGTTACGAGA	59		35	1,500
	OCSP_R4200	TTGGTGTTTTCTTGAAAGTTTTTG				
	OCSP_F4000	TGTCCAAAAGTAGATCCCATGT	57	30		1,400
	OCSP_R5500	CACAAAGGCAAGTTCAAGCA				
	OCSP_F4000	TGTCCAAAAGTAGATCCCATGT	59		35	1,400
	OCSP_R5400	TCCAACATTGCAAATTCGAT				
	OCSP_F5300	GACGAAGAAGGACCCACTTG	57	30		1,000
	OCSP_R3end	AGACGCGAAATGGCATAGAT				
	OCSP_F5300	GACGAAGAAGGACCCACTTG	59		35	1,000
	OCSP_R3stop	GAAGAACTGACGCGGAAAAA				
*Pocsp*	PoCSP_F1	ATGAGGAACTTGGCCATT	50	30		1,100
	PoCSP_R	TTAATGAAAGAATACTAGGAA				
	PoCSP_F2	GCCGTGTCAGCGTTTTTATT	52		35	1,050
	PoCSP_R	TTAATGAAAGAATACTAGGAA				
*Pomsp1*	OMSP1F1	GATGAAATACTAGTCATGGGAA	56	30		1,000
	OMSP1R1	CAT(C/T)ATACTTATCTACTTCCTC				
	OMSP1F1	GATGAAATACTAGTCATGGGAA	58		35	900
	OMSP1R2	CATCATC(A/G)TCTGCGTTTCCC				

### Analysis of variable region in *pomsp1* gene

Twelve available *pomsp1* sequences from both *P*. *ovale curtisi* and *P*. *ovale wallikeri* were retrieved from the NCBI database (accession number FJ824670, FJ824671, KC137340—KC137349) and multiple sequence alignments were performed. A highly polymorphic region within *pomsp1* was observed between amino acid residues 700 to 1,000. The primers OMSP1.F1, OMSP1.R1, and OMSP1.R2, were designed for a semi-nested PCR approach to analyses this polymorphic domain in 25 *P*. *ovale* samples (Table 1). Positive PCR products were then purified by Gel/PCR purification kit (Favogen, Taiwan), before being submitted for DNA sequencing. All *pomsp1* sequences obtained in this study were analyzed together with the previous reports.

### Sequence analysis and phylogenetic tree reconstruction

Nucleotide polymorphisms of *poctrp*, *pocsp* and *pomsp1* from *P*. *ovale curtisi* and *P*. *ovale wallikeri* were analyzed with ClustalW multiple alignment using BioEdit version 7.2.6.1 [24]. Nucleotide sequences of *poctrp*, *pocsp* and *pomsp1* were translated to deduced amino acid sequences using BioEdit version 7.2.6.1 [24]. The sequences obtained from 25 samples of *P*. *ovale* spp. were analyzed in comparison with the previously reported sequences from the NCBI database (*poctrp*: accession number LT594512, LT594589, *pocsp*: accession number SBT72933, SBT84923, *pomsp1*: accession number LT594511, LT594588, KX672044, KX672045, FJ824670, FJ824671, KC137340—KC137349). Genetic variability including average pairwise nucleotide diversity (Pi), haplotype diversity, and sliding plot nucleotide diversity with a window length of 100 bp and 25 bp step size within *poctrp*, *pocsp* and *pomsp1* from *P*. *ovale curtisi* and *P*. *ovale wallikeri* was obtained from DnaSP 6.10.4 [25]. The ratio of non-synonymous to synonymous (dN/dS) within each *P*. *ovale* spp. was measured by DnaSP 6.10.4 [25]. Tests for neutral evolution were assessed with Tajima’s D, Fu and Li’s D, and Fu and Li’s F tests using DnaSP 6.10.4 [25].

A neighbor-joining (NJ) phylogenetic tree was constructed from concatenated CTRP, CSP and MSP1 protein sequences to assess relationships between *P*. *ovale curtisi* and *P*. *ovale wallikeri*. A bootstrap test (1,000 replicates) was applied under the Jones-Taylor-Thornton (JTT) model of evolution using MEGA7 [26].

## Results

### Isolation and analysis of *poctrp*

**Fig 1:**
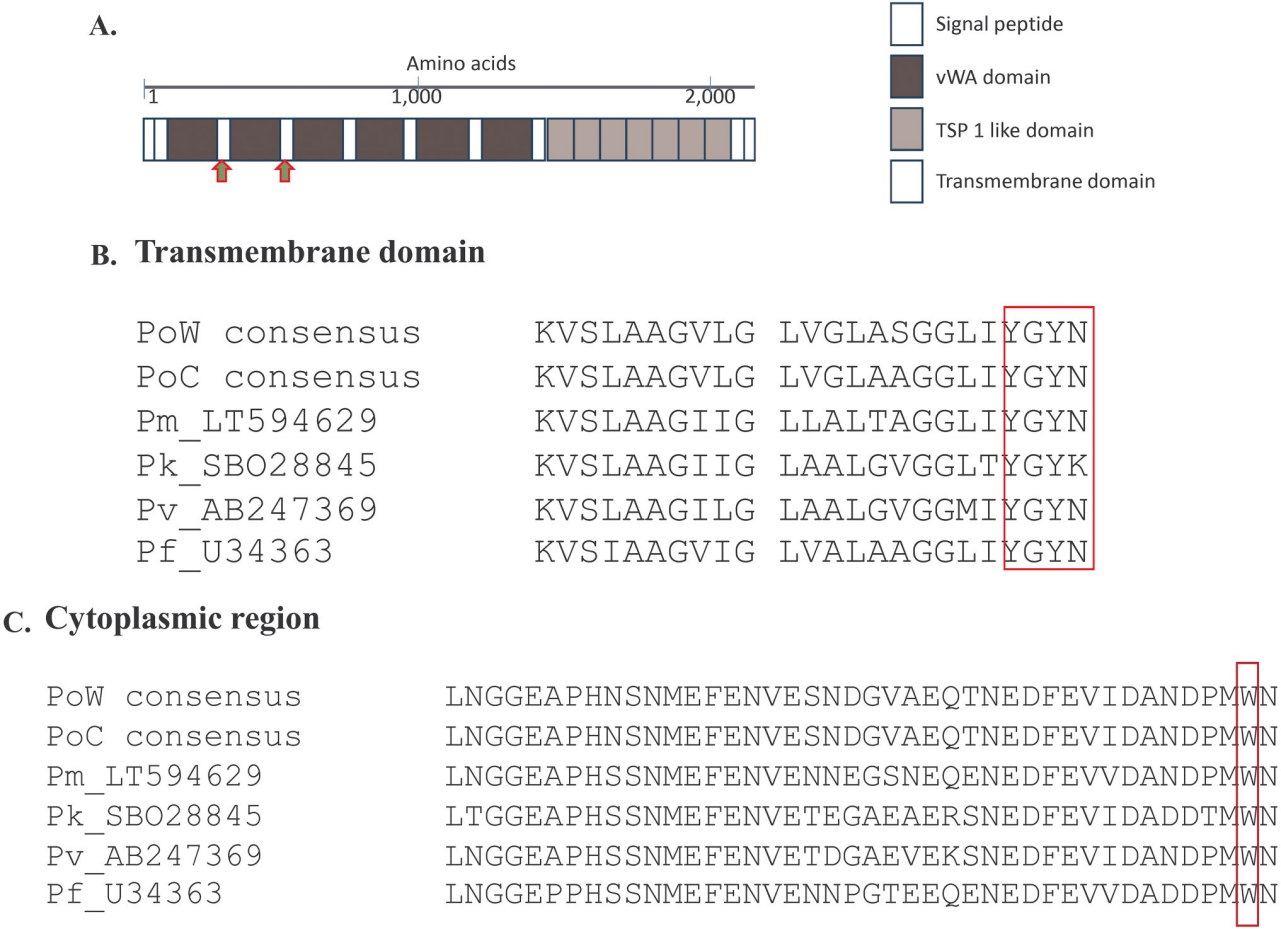
The *Plasmodium ctrp* gene. (A) Schematic representation of the domain structure of *Plasmodium ctrp* gene. The *Plasmodium* CTRP composes of N-terminal signal peptide, six vWA domain, seven tandemly arrayed TSP 1- like domains, and the C-terminal transmembrane domain. Based on *P*. *falciparum* CTRP domain structure analysis, the PoCTRP domains could be drawn from multiple sequence alignment. Amino acid sequence signature for each *Plasmodium* species were observed between vWA 1–2 domains and between vWA 2–3 domains as indicated by red arrows. (B) Amino acid alignment of the transmembrane (TM) domain with the box representing the conserved tyrosine-based motif involved in cellular trafficking. (C) Amino acid alignment of the cytoplasmic region. The conserved tryptophan residue that interacts with motility actomyosin machinery was marked with the box.

The complete coding sequence of *poctrp* gene was obtained from 11 *P*. *ovale curtisi* and 14 *P*. *ovale wallikeri* isolates (accession number MK403987-MK404009). It revealed that the *poctrp* genes for both *species* has only one exon encoding for 2,007 to 2,047 amino acids. Sequence alignment of these 25 *poctrp* sequences, together with another two *poctrp* sequences (accession number LT594512 and LT594589) available in the NCBI database, and other *ctrp* sequences from the other *Plasmodium* spp. that infect humans, showed that *poctrp* is composed of a signal peptide, six vWA domains, seven TSP1 domains, transmembrane domain, and a cytoplasmic region (Fig 1). Alignment of the CTRP of all human *Plasmodium* spp. revealed highly conserved transmembrane (TM) and cytoplasmic regions (Fig 1). A conserved amino acid sequence YGYN/K for the tyrosine-based TM motif involved in cellular trafficking, and the cytoplasmic domain tryptophan residue (Fig 1) which is the key interaction to drive parasite motility were conserved between all studied human *Plasmodium* spp. Multiple alignment of the full-length CTRP among all human-infecting *Plasmodium* spp. also identified a highly conserved region close to the C-terminus.

**Fig 2:**
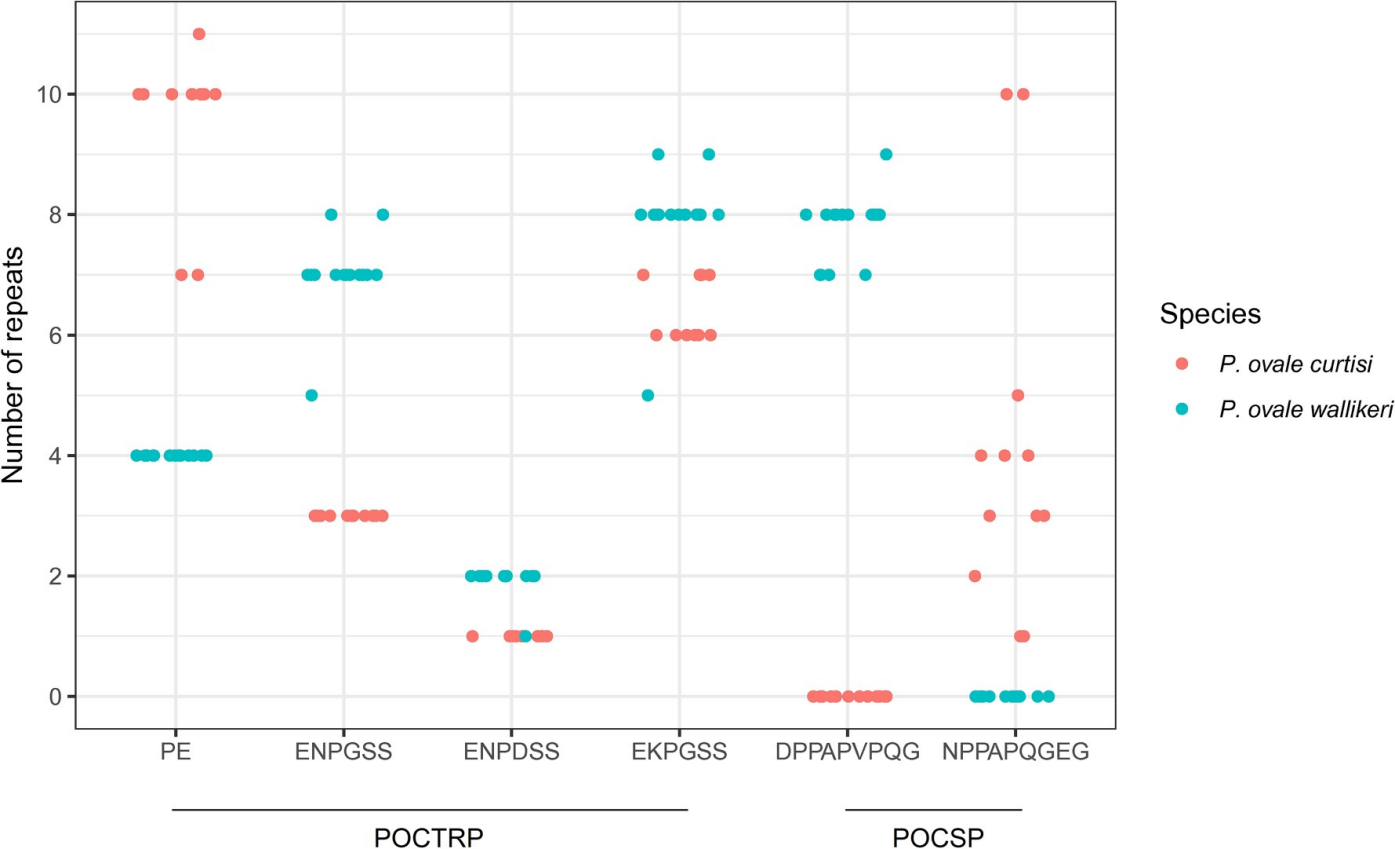
Distribution of the major tandem repeat units in PoCTRP and PoCSP.

All *poctrp* sequences including the two reference sequences were translated to deduce their corresponding amino acids and analyzed for intra- and inter-specific sequence diversity at this locus. The deduced amino acid alignment of PoCTRP showed two prominent regions. The first region is located around 300–320 amino acids between the vWA1 and vWA2 domains. *P*. *ovale curtisi* isolates carries two amino acids repeats “PE” with 7–11 copies, while all *P*. *ovale wallikeri* isolates had 4 “PE” repeat units (Fig 2). The second region is located between codons 570 and 600, where a tandem repeat of six amino acids was identified. Three patterns of six amino acids repeats were observed: ENPDSS, EKPGSS, and ENPGSS. Different numbers of repeat units were presented in the *P*. *ovale* isolates (Fig 2). The repeat EKPGSS is the most frequent in both *P*. *ovale curtisi* and *P*. *ovale wallikeri*. This region showed a marked difference in length, providing a potential additional genotypic marker to differentiate *P*. *ovale curtisi* from *P*. *ovale wallikeri*. Multiple sequence alignment of CTRP of all human *Plasmodium* spp. revealed species-specific regions for *P*. *ovale* spp. at codons 512–538 and codons 573–599. PCR amplification of *P*. *ovale* spp. with primers targeting those two regions are useful to distinguish *P*. *ovale curtisi* from *P*. *ovale wallikeri*.

**Fig 3:**
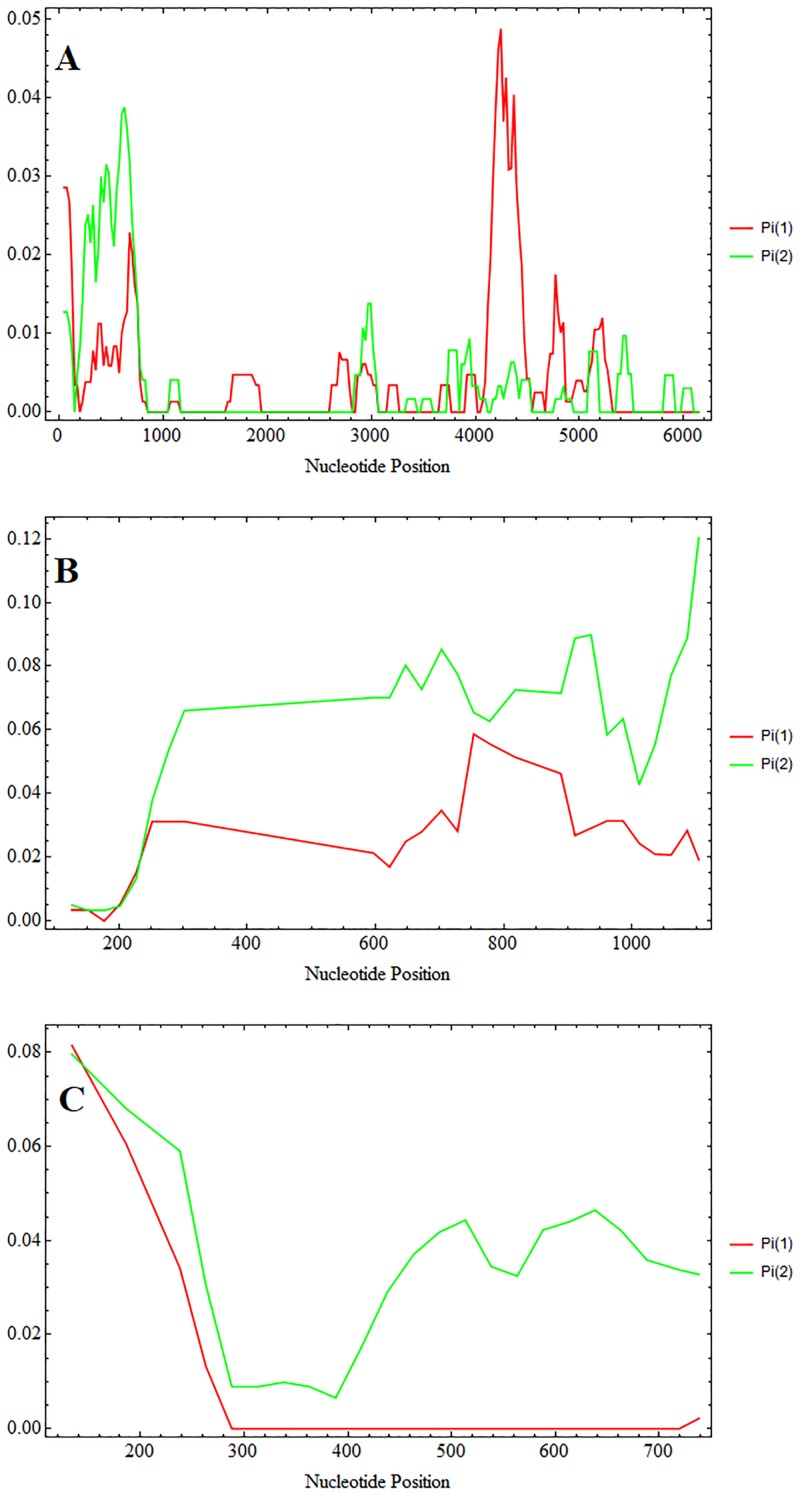
Sliding window plot of nucleotide diversity. Sliding plot with a window length of 100 bp and 25 bp step size using DnaSP v5 revealed nucleotide diversity between *P*. *ovale wallikeri* (Pi 1) and and *P*. *ovale cursiti* (Pi 2). The nucleotide diversity is calculated from *ctrp* (A), *csp* (B), and partial *msp1* gene (C).

The available *poctrp* genes were analysed in a sliding plot for nucleotide diversity between *P*. *ovale wallikeri* and *P*. *ovale curtisi* (Fig 3). *P*. *ovale curtisi* showed higher diversity around the first 1 kb where *P*. *ovale wallikeri* showed higher diversity at 4 kb—5 kb of *poctrp* (Fig 3). For this gene, the average nucleotide diversity of *P*. *ovale curtisi* is slightly lower than that of *P*. *ovale wallikeri*, and combined analysis of all 27 *P*. *ovale* sequences showed a higher diversity value than that calculated from each species alone (Table 2), which indicates distinct distributions of diversity across the *poctrp* locus in the two species.

**Table 2:** Nucleotide diversity and natural selection in *P*. *ovale* spp.

**CTRP**	**Species**	**No. of samples**	**Haplotype diversity**	**Pi**	**dN/dS**	**Tajima's D**	**Fu and Li's D**	**Fu and Li's F**
	*P*. *ovale wallikeri*	15	0.971	0.00473	1.52976	0.17242	0.1958	0.10679
	*P*. *ovale curtisi*	12	1	0.00416	1.11169	0.42001	0.10998	0.03388
	*P*. *ovale*	27	0.991	0.01912	0.28505	2.11247*	1.53105**	2.02216**
**CSP**	**Species**	**No. of samples**	**Haplotype diversity**	**Pi**	**dN/dS**	**Tajima's D**	**Fu and Li's D**	**Fu and Li's F**
	*P*. *ovale wallikeri*	15	0.93333	0.02529	0.47695	1.08735	0.99957	1.1802
	*P*. *ovale curtisi*	12	1	0.05958	0.88465	0.588	0.82928	0.87353
	*P*. *ovale*	27	0.98006	0.1201	0.96289	1.45552	1.4117*	1.68304*
**MSP1**	**Species**	**No. of samples**	**Haplotype diversity**	**Pi**	**dN/dS**	**Tajima's D**	**Fu and Li's D**	**Fu and Li's F**
	*P*. *ovale wallikeri*	20	0.511	0.01309	2.24763	1.28828	1.71084**	0.94791
	*P*. *ovale curtisi*	21	0.867	0.03582	1.32754	0.80269	0.78703	0.92536
	*P*. *ovale*	41	0.817	0.11226	0.89189	1.79705	1.58881**	1.98472**

* P<0.05,

**P<0.02

### Isolation and analysis of *pocsp*

The *pocsp* gene was successfully amplified from 14 *P*. *ovale wallikeri* and 11 *P*. *ovale curtisi* isolates (accession number MK404010-MK404031). The complete *pocsp* gene varied in size from 1,020 to 1,185 bp, and the size variation resulted from variable tandem repeats in the central repeat region. The *pocsp* sequences were analyzed together with two other sequences available in NCBI databases and those of the *csp* of the other human *Plasmodium* spp. The protein domain architecture of *pocsp* was determined based on homologous CSP proteins alignment with other human *Plasmodium* spp. The *pocsp* structure domain was similar to that of the *csp* from the other *Plasmodium* spp. Four domains in the conserved N-terminus domain (conserved region I) and in the conserved C-terminus domain (Th2R, conserved region II, and Th3R) were of particular interest. A summary of the amino acid patterns in each of these domains is presented in Table 3. Overall, a higher number of haplotypes was observed in *P*. *ovale curtisi* as compared to *P*. *ovale wallikeri*. Whereas *P*. *ovale wallikeri* showed only one haplotype in conserved region I and two in conserved region II, three and six, respectively, were observed for *P*. *ovale curtisi*. A high number of haplotypes was observed in the Th2R and Th3R domains for both *P*. *ovale curtisi* and *P*. *ovale wallikeri*, but it is interesting to note that none were shared by both species. The central repeat region of *pocsp* was also analyzed. Several patterns of nine amino acid repeats were observed. A specific repeat unit was observed for *P*. *ovale wallikeri* (DPPAPVPQG), and for *P*. *ovale curtisi* NPPAPQGEG, with the latter showing a higher diversity in repeat unit numbers (Fig 2).

**Table 3:** Sequence polymorphism in the conserved regions of *P*. *ovale* CSP.

Haplotype	Amino acid	No. of *P*. *ovale curtisi*	No. of *P*. *ovale wallikeri*
	**Conserved region I**		
1	PVENKLKQG	6	15
2	PVENKLNQG	1	0
3	PVENNLNQG	5	0
	**Th2R**		
1	PPSEDDIKKYIDKIRKD	0	7
2	PPSEDDIKKYIDKIRND	2	0
3	PPSEDDIKKYIDKIRRD	0	1
4	PPSEDDIKKYLDKIRRD	0	1
5	PPSEDDIKKYLDRIRKD	0	1
6	PPSEDDIKNFIDKIRND	1	0
7	PPSEDDIKRYLDRIRND	1	0
8	PPSEDDIKSFIDKIRND	3	0
9	PPSEDDIRKYIDKIRRD	0	1
10	PPSEDDIRRYLDKIRND	1	0
11	PPSEDDIRSFIDKIRND	1	0
12	PPSEDDLKKFLDKIRRD	0	2
13	PPSENDIKSFLDKIRND	1	0
14	PPSENDIKSFMDKIRND	1	0
15	PPSENDIRKYIDRIRKD	0	1
16	PPSENDIRNFIDKIRND	1	0
17	PPSENDLKKFLDKIRRD	0	1
	**Conserved region II**		
1	ITENWSPCRVTCG	0	5
2	ITENWSPCSVSCG	1	0
3	ITENWSPCSVSCV	2	0
4	ITENWSPCSVTCG	4	10
5	ITENWSPCSVTCV	1	0
6	LTENWSPCSVSCG	2	0
7	LTENWSPCSVTCG	2	0
	**Th3 R**		
1	KKAGANAKKAQKFTLSDLE	1	0
2	KKAGANAKKAQKLTLSDFE	1	0
3	KKAGANAKKGQKFTLSDFE	1	0
4	KKAGASAKKANELPINDVE	0	3
5	KKAGASAKKANELTINDVE	0	5
6	KKAGASAKKAPKFTLSDLE	1	0
7	KKAGASAKKAQELTLSDLE	3	0
8	KKAGASAKKAQKFTLSDLE	1	0
9	KKAGASAKKGPKLTLSDLE	1	0
10	KKAGASAKKGQKFTLSDLE	1	0
11	RKAGASAKKANELPINDVE	0	1
12	RKAGASAKKANELTINDVE	0	6
13	RKAGASAKKAQELTLSDLE	2	0

The *pocsp* gene was evaluated for nucleotide diversity between *P*. *ovale curtisi* and *P*. *ovale wallikeri*. Sliding plots of nucleotide diversity revealed overall higher nucleotide diversity in *P*. *ovale curtisi* than in *P*. *ovale wallikeri* (Fig 3). The estimated synonymous (dS) and nonsynonymous (dN) substitution was also found at higher value in *P*. *ovale curtisi* than that of *P*. *ovale wallikeri* (Table 2). Combined analysis of both *P*. *ovale* spp. showed significantly positive values (p<0.05) for Fu and Li’s D and Fu and Li’s F tests, suggesting population bottlenecks or balancing selections in these two species (Table 2).

### Genetic analysis of *pomsp1*

The sequences for the variable regions within the *pomsp1* gene covering amino acids 710 to 1,020 were obtained from 14 *P*. *ovale wallikeri* and 11 *P*. *ovale curtisi* isolates (accession number MK404032-MK404049). Apart from this, sixteen sequences of *pomsp1* gene (accession number LT594511, LT594588, KX672044, KX672045, FJ824670, FJ824671, KC137340—KC137349) were available in the NCBI database. Taken together, 41 PoMSP1 sequences were used in the alignment. A clear dimorphic pattern was observed between *P*. *ovale curtisi* and *P*. *ovale wallikeri*. Amino acid tandem repeat patterns were found in *P*. *ovale* spp. The tandem repeats are characteristic for the two different *P*. *ovale* spp. There were three arrangement patterns of three 5-amino acid repeat units (PGAGG, PGAAG, and PGVPG) found exclusively in *P*. *ovale wallikeri* isolates Whereas, nine arrangement patterns of six 4-amino acids repeat units (QAAT, QTAT, HAST, QATT, QVTT, QSAT) were observed specifically in the *P*. *ovale curtisi* isolates (S2 Table).

Analysis of gene diversity and haplotype diversity at the *pomsp1* locus showed that *P*. *ovale curtisi* has higher diversity than that of *P*. *ovale wallikeri* (Table 2). Sliding window plots showed higher overall nucleotide diversity in *P*. *ovale curtisi* than in *P*. *ovale wallikeri* (Fig 3). The ratio of synonymous (dS) and nonsynonymous (dN) substitutions was higher in *P*. *ovale wallikeri* than *P*. *ovale curtisi* (Table 2).

### Comparative analysis of *P*. *ovale curtisi* and *P*. *ovale wallikeri*

Genetic analysis of *P*. *ovale curtisi* and *P*. *ovale wallikeri* based on three surface protein genes revealed clear dissociation between these two species. Analysis within each species was performed though sequence diversity and amino acid patterns. The sequence polymorphism in *poctrp*, *pocsp* and *pomsp1* showed more divergence in *P*. *ovale curtisi* than in *P*. *ovale wallikeri* (Fig 3, Table 2). The test for neutrality (Tajima’s D, Fu and Li’s D, and Fu and Li’s F tests) was applied to *poctrp*, *pocsp* and *pomsp1* to compare observed polymorphism frequencies with expected frequencies. Significantly positive values were obtained from Fu and Li’s D and Fu and Li’s F when *P*. *ovale* spp. were analyzed as one group (Table 2). These statistics reflect higher than expected frequencies of alleles, which might have resulted from population bottlenecks or balancing selections. *P*. *ovale curtisi* had a higher number of different haplotypes in all conserved domains of the CSP (Table 3). This suggests that *P*. *ovale curtisi* is intrinsically more genetically diverse than *P*. *ovale wallikeri*, but may also represent limitations of our sample.

Some of the studied *P*. *ovale curtisi* and *P*. *ovale wallikeri* infections were mixed with other human malaria spp., which might have impacted the characteristics of CTRP, CSP and MSP1. Therefore the genetic analysis of CTRP, CSP and MSP1 was compared between single and mixed infections. There were four mixed infection found in *P*. *ovale wallikeri*, in which all four samples were collected from Thailand. For *P*. *ovale curtisi*, most samples were collected from Africa with four single infections and five mixed infections. There was no significant difference in average nucleotide diversity (Pi), haplotype diversity, and dN/dS between single and mixed infections. Statistical testing for neutrality (Tajima’s D, Fu and Li’s D, and Fu and Li’s F tests) was also not significantly different (S3 Table). In addition, the pattern of tandem repeats in CTRP, CSP and MSP1 in *P*. *ovale* spp. showed no difference either between single and mixed infections or between Asia and Africa isolates.

**Fig 4:**
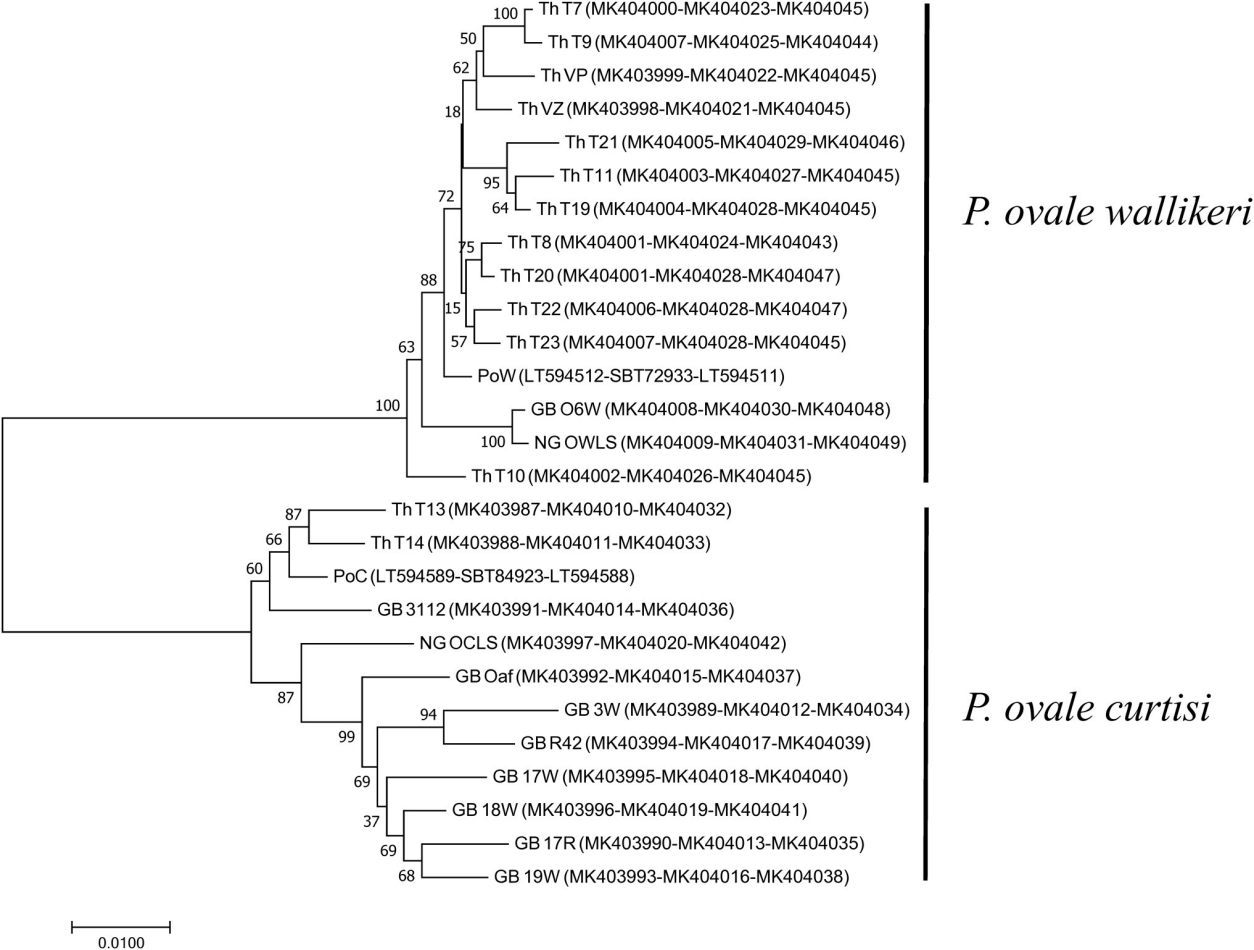
Phylogenetic analysis of *P*. *ovale* spp. Phylogenetic tree inferred using the Neighbor-Joining method based on concatenated CTRP, CSP, and MSP1 proteins. The percentage of replicate trees in which the associated taxa clustered together in the bootstrap test (1,000 replicates) are shown next to the branches. The evolutionary distances were computed using the JTT matrix-based method and are in the units of the number of amino acid substitutions per site. Accession numbers of CTRP-CSP-MSP1 of each sample is shown in the bracket.

To infer genetic relationships of *P*. *ovale curtisi* and *P*. *ovale wallikeri*, a phylogenetic tree was reconstructed based on the three cell-surface associated proteins, CTRP, CSP and MSP1. The Neighbour-Joining method [27] was used to infer the evolutionary history of each species. Based on CTRP, CSP and MSP1 (Fig 4), *P*. *ovale curtisi* and *P*. *ovale wallikeri* clustered according to species, and the tree topologies inferred from each gene showed similar features of grouping into *P*. *ovale curtisi* and *P*. *ovale wallikeri*. This suggested that the genes of *P*. *ovale curtisi* and *P*. *ovale wallikeri* do not recombine and show distinct characteristics (Fig 4).

## Discussion

In addition to the earlier described polymorphisms in *pomsp1*, the current study provides the genetic characterization for two more cell-surface associated proteins: *ctrp* and *csp*. Analysis of the complete *ctrp* gene from all human *Plasmodium* spp., including the *P*. *ovale* species presented here, revealed a strongly conserved region in the CTRP protein, likely related to its importance for parasite survival. The highly conserved transmembrane and cytoplasmic regions are likely associated with cellular trafficking and parasite development. The C-terminal part containing residues and domains crucial for CTRP function for all human *Plasmodium* spp. were also conserved. This protein could therefore be a candidate target for vaccine development. Analysis of the *csp* gene in *P*. *ovale curtisi* and *P*. *ovale wallikeri* revealed a similar gene structure compared to that of the other human malaria species. The amino acid haplotypes observed in the conserved region of the *csp* gene were nearly all specific to either one or other species, with only 1/3 and 1/7 shared for conserved region I and conserved region II, respectively. Interestingly, no overlap was observed for the 17 Th2R and 13 Th3R haplotypes detected. This could imply a species-specific immune interactions with these T helper epitopes, or indicate a distinct biologically functional constraint. As the two species harbor distinct *pocsp* repeat regions, NPPAPQGEG and DPPAPVPQG, respectively, these peptides may provide useful species-specific targets for the development of antibody reagents for serological distinction of sporozoites from the two ovale species.

The study also provided addition information on the cell-surface associated protein, MSP1. Analysis of the variable region within *pomsp1* of 25 *P*. *ovale* samples in this study supplemented with 16 *pomsp1* from previous reports showed a clear distinction between *P*. *ovale curtisi* and *P*. *ovale wallikeri*. Alignment of the MSP1 from all human *Plasmodium* spp. showed the interspecies conserved blocks corresponding to previous characterizations [28]. Sequence polymorphisms of CTRP, CSP and MSP1 from each *P*. *ovale* spp. can be used for determination of parasite evolutionary relationships. Phylogenetic tree reconstruction based on concatenated CTRP, CSP and MSP1 clearly showed that *P*. *ovale curtisi* and *P*. *ovale wallikeri* are cluster separately, consistent with previous reports [29, 30].

Analogies in the reported surface proteins in *P*. *ovale* with other human *Plasmodium* species could help selecting potential vaccine candidates. For instance, CTRP affects oocyst development of *P*. *falciparum* in *Anopheles* mosquitoes [10], and conserved regions within CTRP across human *Plasmodium* spp. could provide candidate targets for transmission-blocking vaccine. In MSP1, domain architectures are similar between all human *Plasmodium* spp., and our study of PoMSP1 revealed an interspecies conserved domains 6 (residues 812–911) between the *Plasmodium* spp., which could be candidates for a trans-species malaria vaccine. Our data could also provide the basis for development of new serological reagents for distinguishing the two species, and for identifying individuals with a history of exposure to *P*. *ovale* spp. carrying species-specific serum antibodies. In addition to the genes evaluated in this study, other important polymorphic genes have been used for discrimination between the two *P*. *ovale* spp., including the *surfin* variant gene family and the *Plasmodium* interspersed repeat (*pir*) superfamily, which showed expansion in both *P*. *ovale* spp.[7]. Additional genes encoding potential targets for vaccine development warrant further study, including genes encoding reticulocyte binding proteins and tryptophan-rich domains [31].

In summary, this study showed conserved domains in the *poctrp* and *pocsp* genes which code for potential targets for future vaccines. Quantifying polymorphism in nucleotide sequences and the tandem repeat diversity between *P*. *ovale curtisi* and *P*. *ovale wallikeri* showed absence of recombination, supporting their designation as distinct species. Within the three analysed genes, diversity was higher in *P*. *ovale curtisi* than in *P*. *ovale wallikeri*. However, this will need to be confirmed in a larger sample size with better comparison between the geographical areas where the strains were collected. In the current sample most *P*. *ovale curtisi* was collected from highly endemic African countries whereas most *P*. *ovale wallikeri* were collected in Thailand which has low endemicity.

## Supporting information

S1 Table List of samples used in the study. (XLSX)

S2 Table Amino acid pattern of partial MSP1 in *P*. *ovale* spp. (XLSX)

S3 Table Comparative analysis of single and mixed infections *P*. *ovale* spp. (XLSX)
